# Predicting the Tensile Behavior of Ti-6.6Al-3.3Mo-1.8Zr-0.29Si Alloy via the Temperature-Dependent Crystal Plasticity Method

**DOI:** 10.3390/ma12193138

**Published:** 2019-09-26

**Authors:** Jun Zhang, Yang Wang, Peng Wang, Junhong Chen, Songlin Zheng

**Affiliations:** 1 Institute of Systems Engineering, China Academy of Engineering Physics, Mianyang 621999, China; hjwangp@caep.cn (P.W.); lxchenjh@caep.cn (J.C.); 2 CAS Key Laboratory of Mechanical Behavior and Design of Materials, Department of Modern Mechanics, University of Science and Technology of China, Hefei 230027, China; 3 National Key Laboratory of Shockwave Physics and Detonation Physics, Institute of Fluid Physics, China Academy of Engineering Physics, Mianyang 621900, China; slzheng@caep.cn

**Keywords:** titanium alloy, duplex microstructure, crystal plasticity, work-hardening, temperature dependency

## Abstract

Uniaxial tensile flow properties of a duplex Ti-6.6Al-3.3Mo-1.8Zr-0.29Si alloy in a temperature range from 213 K to 573 K are investigated through crystal plasticity modelling. Experimental results indicate that the initial yield stress of the alloy decreases as the temperature increases, while its work-hardening behavior displays temperature insensitivity. Considering such properties of the alloy, the dependence of the initial critical resolved shear stress (CRSS) on temperature is taken into account in the polycrystal plasticity modelling. Good coincidence is obtained between modelling and the experimental results. The determined values of CRSS for slip systems are comparable to the published data. The proposed polycrystalline model provides an alternative method for better understanding the microstructure–property relationship of α + β titanium alloys at different temperatures in the future.

## 1. Introduction

α + β titanium alloys, especially the alloys containing a lamellar microstructure, have been attractive structural metals among the military and civil fields due to their comprehensive advantages, e.g., high specific strengths, high resistance to corrosion, and high-damage tolerant properties [1]. Nowadays, the mechanical behavior of such titanium alloys has been improved via controlling the microstructures and alloying elements [2,3]. Since these alloys inevitably encounter large deformations when servicing over a wide range of temperatures, the temperature-dependent elastoplastic feature is one of the important issues for the structure design. Thus, it is essentially required to predict the temperature-dependent response of the alloys with various microstructures.

Polycrystal plasticity methods have been an efficient tool to study the microstructure-dependent properties of the alloys [4]. For polycrystalline modelling, one challenge is how to consider more abundant microstructural information in the model, such as texture, grain size, and shape [5,6]. Another challenge is how to simulate the competitive behavior between multiple mechanisms under different strain rates and temperatures [7,8,9,10]. Currently, several crystal plasticity finite element models (CPFEM) have been conducted on several α + β titanium alloys containing a lamellar microstructure. The difficulty of polycrystalline modelling for this kind of structure lies in the explicit description of the α + β colony structure, since the thickness of lamellar β grain has been received as many times smaller than those of other grains in lamellar α + β titanium alloys. Thus, the isostrain [11,12,13,14] or isostress [15,16,17] assumptions of the lamellar α + β colony have been proposed. Compared with other explicit modelling of α + β colony structure [18,19], the application of the equivalent homogenized technique makes it possible to conduct an efficient simulation on the behavior of polycrystalline α + β titanium alloys containing a huge number of grains. For example, Ghost and his co-workers [11,12,13,14] established a series of experimentally validated polycrystalline models of duplex α + β titanium alloys. McDowell et al. [15,16,17] investigated the cyclic deformation of a duplex Ti-6Al-4V alloy at room temperature using a homogenized CPFE model. Fan et al. [20,21] also employed a similar model to study the morphological effect on the flow softening behaviour of titanium alloys at high temperatures, from 1088 to 1228 K.

Nevertheless, the aforementioned microstructure-based crystal plasticity models mainly deal with the deformation features at room temperature or at high temperatures over 1000 K. Little research has been publicly reported about the numerical simulation for duplex α + β titanium alloys at low to medium temperatures. In fact, temperature plays a critical role on the elastoplastic feature in the single-crystal, e.g., the initial critical resolved shear stress (CRSS) of the dislocation slips in the α/β grains. As demonstrated in previous studies by Williams et al. [22], values of CRSS for basal and prism slips in α grains decrease with increasing temperature in the range of 77–1000. The CRSS in a single α + β titanium colony at high temperature was also experimentally verified to be much lower than that at room temperature [23]. Thus, it is worth further exploring the temperature-dependent polycrystalline model of duplex α + β titanium alloys in the low to medium temperature region.

The Ti-6.6Al-3.3Mo-1.8Zr-0.29Si alloy has become a prospective α + β type titanium alloy for aero-engine turbo blades. The effects of strain rate and temperature on the constitutive responses of the alloy have been widely investigated through the phenomenological models [24,25,26]. Since these conventional constitutive models do not account for the detailed microstructure feature, they lack guidance for the property optimization. To further promote the properties of the alloy, the objective of this paper is to develop a microstructure-based crystal plasticity model and study the temperature-dependent flow behavior of the Ti-6.6Al-3.3Mo-1.8Zr-0.29Si alloy. The paper is organized as follow: in Section 2, the quasi-static deformation features at temperatures of 213–573 K are introduced. In Section 3, temperature-dependent CRSS, as well as equivalent homogenized technique, are employed to establish the numerical CPFE model of polycrystalline alloy. Finally, model parameters are determined and the reasonability of the polycrystalline model is discussed.

## 2. Deformation Features at Various Temperatures

**Figure 1:**
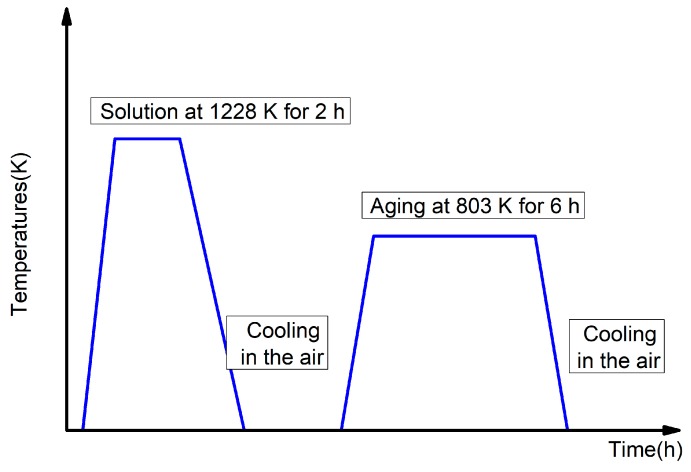
Scheme of double heat treatment process.

**Figure 2:**
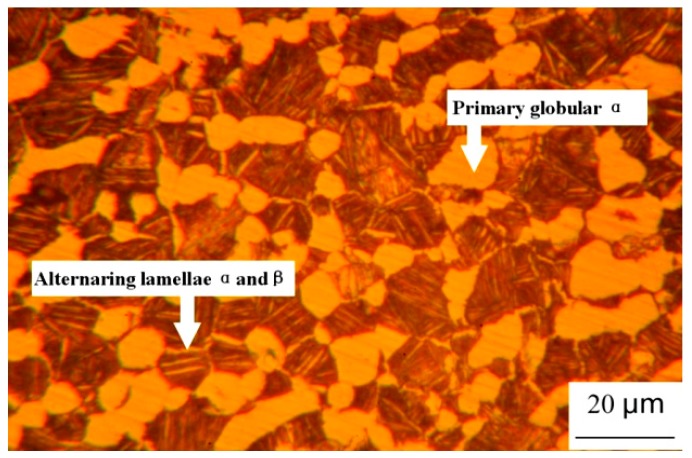
The microstructure of the undeformed specimen [24].

The investigated metal is duplex Ti-6.6Al-3.3Mo-1.8Zr-0.29Si alloy (Chinese band TC11). The duplex microstructure was obtained through the double heat-treatment method as shown in Figure 1. The solution and aging temperatures were 1228 K and 803 K, respectively. Figure 2 depicts the corresponding microstructure of the undeformed specimen, which was a mixture of alternating lamellar α + β colonies and globular α matrix [24]. The volume percentage and dimension of globular α matrix were determined as approximately 45% and ten microns, respectively. The average size of α + β colonies was measured as about twenty microns.

**Figure 3:**
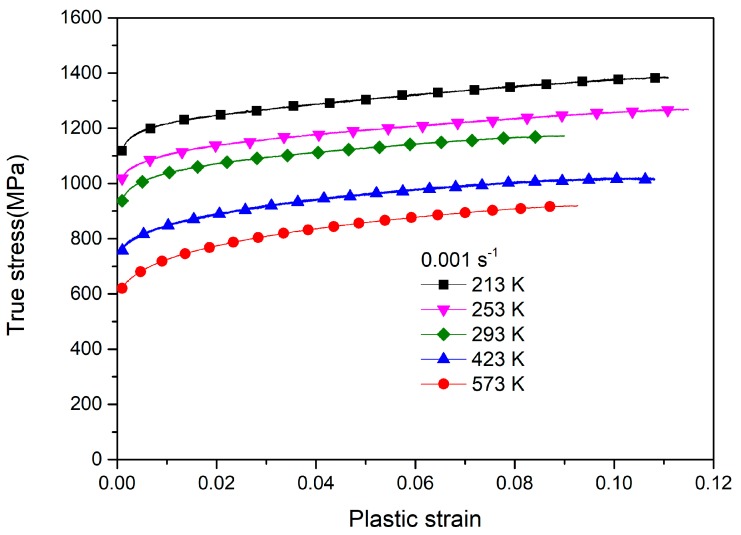
The variation in flow curves at different temperatures.

Figure 3 shows the flow curves of duplex TC11 at a rate of 0.001 s^−1^ and temperatures of 213–573 K. As seen in Figure 3, the alloy shows a nonlinear work-hardening characterization. The initial yield stress (adopted as the stress at 0.2% plastic strain) decreased with the increasing temperature, indicating the obvious temperature softening effect on the yield behavior. Meanwhile, the flow curves after initial yielding at five temperatures are almost parallel, revealing that the work-hardening behavior exhibits little dependence on the temperature from 213 K to 573 K. Such flow features are also consistent with those observed in Ti-6Al-4V alloys below 600 K [3,27,28].

**Figure 4:**
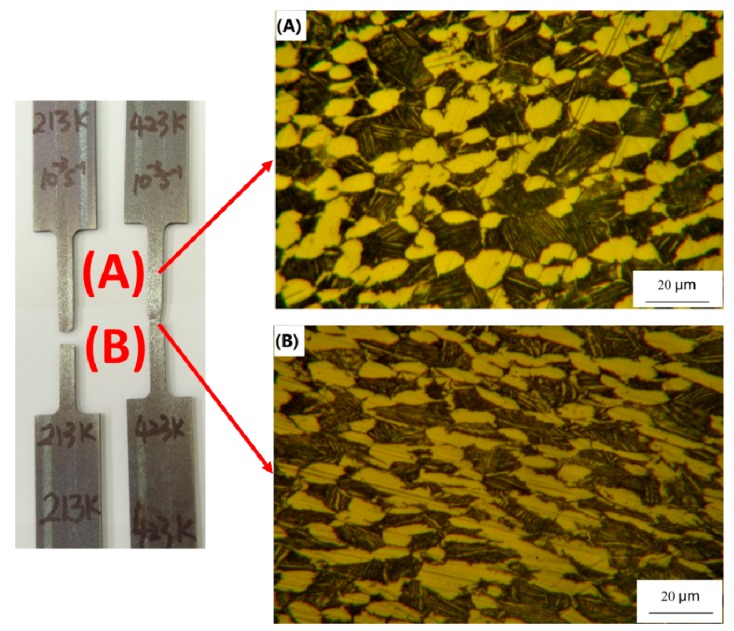
The deformed microstructure: (**A**) the uniform deformation zone; (**B**) the localized necking area.

Figure 4 displays the corresponding microstructures in the uniform deformation zone and localized necking area, marked via symbol (A) and (B), respectively. The extension of the α grains and the α + β colonies are visibly observed. These results of microstructural observation suggest that the macroscopic flow feature is indeed related to the elastoplastic deformation of all constituent grains in the α + β titanium alloy.

## 3. Model Set Up

### 3.1. The Classical Crystal Plasticity Framework 

As stated above, the work-hardening behavior of duplex Ti-6.6Al-3.3Mo-1.8Zr-0.29Si alloy exhibited temperature insensitivity. The crystal plasticity simulation was employed here to model such temperature-dependent flow behavior. The details of the corresponding simulation are presented below. 

The adopted kinematic framework of crystal plasticity theory has been outlined by other literature [29]. The elastic constitutive law is depicted as follows:

(1) $$T=C:E_{e}\;E_{e}=1/2[{F_{e}}^{T}F_{e}-I]T=(\det F_{e})F_{e}{}^{-1}\sigma F_{e}{}^{-T},$$

where **T** is the second Piola–Kirchhoff stress tensor and **σ** is the Cauchy stress tensor. **C** represents the anisotropic elastic tensor and **E**_e_ denotes the Green–Lagrangian strain tensor. **F**_e_ ($F_{e}=FF_{p}{}^{-1},\det F_{e}>0$) is the elastic deformation gradient, and **F** and **F_p_** represent the deformation gradient and its plastic component.

The flow velocity gradient related to the slip is expressed as:

(2) $$L_{p}={\dot{F}}_{P}{F_{P}}^{-1}=\sum_{i=1}^{n}{\dot{\gamma }}^{\left(i\right)}S^{\left(i\right)}n^{\left(i\right)},$$

where ${\dot{\gamma }}^{\left(i\right)}$ is the plastic shear rate of *i*th slip. $S^{\left(i\right)}$ and $n^{\left(i\right)}$ are the corresponding slip plane and slip direction in global coordinate system.

The famous simple power form was used to describe the plastic shear rate, which is given by:

(3) $${\dot{\gamma }}^{\left(i\right)}=\overset{}{{\dot{\gamma }}_{0}^{\left(i\right)}}{\left(\left|\frac{\tau^{\left(i\right)}}{\tau_{c}{}^{\left(i\right)}}\right|\right)}^{\frac{1}{m}}\mathrm{sgn}(\tau^{\left(i\right)}),$$

where ${\dot{\gamma }}_{0}^{\left(i\right)}$ and m are the reference plastic shear rate and rate sensitivity parameter of the material, respectively. $\tau^{\left(i\right)}$($\tau^{\left(i\right)}=n_{}^{\left(i\right)}TS_{}^{\left(i\right)}$) is the resolved shear stress applied on the *i*th slip. $\tau_{c}{}^{\left(i\right)}$ represents the slip resistance. When time is zero, the value of $\tau_{c}{}^{\left(i\right)}$ is equal to the CRSS $\tau_{0}{}^{\left(i\right)}$.

The evolution of deformation resistance $\tau_{c}{}^{\left(i\right)}$ was formulated here by:

(4) $$\overset{}{{\dot{\tau }}_{c}{}^{\left(i\right)}}=h_{0}{}^{\left(i\right)}\sec h^{2}\left|\frac{h_{0}{}^{\left(i\right)}\gamma }{\tau_{s}{}^{\left(i\right)}-\tau_{0}{}^{\left(i\right)}}\right|(\sum_{j}\left[q_{1}+(1-q_{1}\right)\delta_{ij}]\overset{}{{\dot{\gamma }}^{\left(j\right)}},\;\gamma =\sum_{i=1}^{N+M}\int_{0}^{t}\left|\gamma^{\left(i\right)}\right|dt,$$

where $h_{0}{}^{\left(i\right)}$ represents the initial hardening modulus. $q_{1}$ and γ are the latent hardening constant and the total shear strain, respectively. $\tau_{s}{}^{\left(i\right)}$ represents the saturation flow stress. 

### 3.2. Temperature Dependence of CRSS

As stated above, the initial yield stress of the TC11 alloy decreases with an increase in temperature. Considering such deformation characteristics, the initial critical shear stress τ_0_ was treated as temperature-dependent, while h_0_ and τ_s_ were assumed to be independent of the temperature due to the temperature-independent strain-hardening. This simplification is different from the other temperature-dependent theories [7,8,9,10]. Normally, the CRSS is decomposed into the sum of the athermal term $s_{a}^{\left(i\right)}$, related to the long-range barriers and the thermal-dependent part due to the short barriers. Here, the temperature dependency of CRSS was introduced through:

(5) $$\left|\tau_{0}{}^{\left(i\right)}(T)\right|=s_{a}^{\left(i\right)}+\overset{\wedge }{\tau^{\left(i\right)}}{\left[1-{\left(\chi \frac{k_{B}T}{\Delta G_{0}{}^{\left(i\right)}}\right)}^{1/q}\right]}^{1/p},$$

where $\overset{\wedge }{\tau^{\left(i\right)}}$ is the absolute resistance at 0 K. $\Delta G_{0}{}^{\left(i\right)}$ represents the activation enthalpy and $k_{B}$ is Boltzmann constant. χ, p, and q are the thermal-activated relative constants.

### 3.3. Construction of the Duplex Microstructure

The adopted simulation model here followed the representative elementary volume model of polycrystalline magnesium [9,10] and the polycrystalline methods of duplex Ti-6Al-4V [11,12,13,14,15,16,17]. In the present numerical model, grain shape was approximately handled through cubic blocks and each cubic block represented either an α grain or a lamellar α + β colony. The equivalent homogenized method was utilized to calculate the mechanical behavior of transformed α + β colonies [15,16,17]. The adjacent grains were perfectly connected—the grain boundary was ignored. The distinct elastoplastic deformation was described by the aforementioned crystal plasticity constitutive formulations. The final simulation was conducted through the commercial finite element platform using the user-defined material routine, in which the detail implementation equations and integration scheme were presented in the literature by Huang et al. [29].

**Figure 5:**
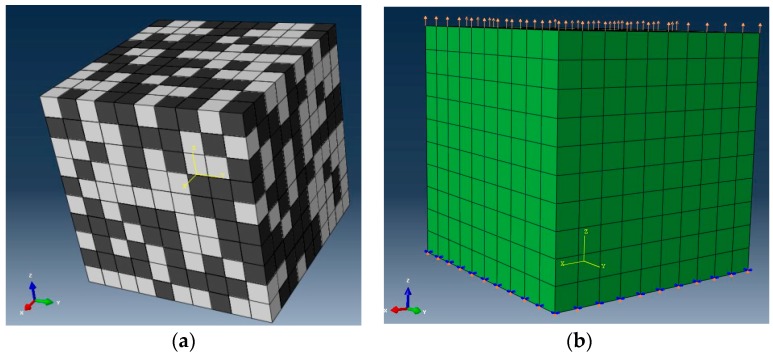
The numerical model of duplex α + β titanium alloy, where dark means a primary α grain and the other is the α + β colony. (**a**) The representative elementary volume; (**b**) the boundary conditions.

The specimen meshed by eight-node brick elements was constructed to simulate the stress–strain behavior of alloys. In order to eliminate fluctuation error, a total of 1000 hybrid grains consisting of globular α grain and lamellar α + β clusters were employed. The orientation and the distribution of each α grain or a transformed α + β colony were initialed by the Bunge Euler angles in the Cartesian configuration [30]. A random texture was initialed based on the actual fraction of the constituent phase. As demonstrated in Figure 5, one end of the model was an applied symmetric boundary and the velocity boundary was applied on the other end, where the force and displacement were outputted to simulate the macroscopic true stress–strain behavior at constant strain rate conditions.

Dislocation slip has been recognized as dominant plastic mechanism in Ti-Al alloys [22,23,31,32,33,34,35]. Twinning commonly occurs in titanium alloys with low Al content [22]. It was noticed that the Al content in the TC11 alloy was high up to 6.6%. Therefore, the twinning mechanism was ignored in the present model and only the dislocation mechanism was considered here in the model. The globular α phase includes the basal and prismatic slip with $<11\bar{2}0>$, as well as the first-order pyramidal slip with $<11\bar{2}3>$. The lamellar α + β colony includes {110}<111> slip systems corresponding to the bcc-structured β phase, and prismatic and basal $<11\bar{2}0>$ slip corresponding to hcp secondary α grains. The deformation along the c-axial direction can be provided by the first-order $\{10\bar{1}1\}$ $<11\bar{2}3>$ in the primary α grain and the {110}<111> slip systems corresponding to α + β colony regions.

### 3.4. Determination of Material Parameters

Table 1 lists the adopted material parameters in the model. The globular α grains, as well as lamellar α + β aggregates, were simply treated as the transversely isotropic material. The five parameters and their dependence on the temperature were approximately given by the following expression [20,21]:

(6) $$C(T)=C^{\mathrm{RT}}\frac{\mu (T)}{\mu_{\mathrm{RT}}};$$

(7) μ(T)=49.02−5.821/[exp(181/T)−1];

where μ(T) is the macroscopic shear modulus at temperature *T*. For the TC11 alloy, the value of the shear modulus at room temperature, $\mu_{\mathrm{RT}}$, was measured as about 42 GPa. Considering the little difference among the elasticity module, values of ${C_{ij}}^{\mathrm{RT}}$ used here were taken from the literature for the Ti-6Al-4V alloy [15].

The other parameters were related to the plastic slip behavior. Here, the reference strain rate in equation 4 was taken as 0.001 s^−1^. On the basis of the studies by Waheed et al. [18], the rate sensitivity constant, *m*, for all the slip systems was adopted as 0.02. For the latent hardening behavior, the values of parameter q_1_ were suggested to be between 1 and 1.4 [4]. Here, the adopted value of q_1_ was 1.0. Referring to the values used in other HCP and BCC structured metals [8], *p* and *q* in Equation (5) were adopted as 0.5 and 2 in the α grains, while they were 0.5 and 1.25 for the β grains. 

The temperature dependence of CRSS was first evaluated by fitting Equation (5) with the experiment data for a single Ti-6.6Al crystal [22]. The values of CRSS were constrained according to the published data for α + β polycrystalline titanium alloys [11,12,13,14,15,16,17] and a single α crystal or single α + β colony [22,23,31,32,33,34,35]. The CRSS ratio of the slip families in primary α grains was set to be $1:\varsigma_{1}:\varsigma_{2}$ for prismatic<a>, basal<a>, and first-order pyramidal<c + a>, where $\varsigma_{1}$ varied between 1 and 1.2 and $\varsigma_{2}$ was taken from 2.5 to 5 [15]. The slip families in lamellar α + β colonies were divided into the soft model and the hard model based on the previous investigations [13,15]. The CRSS values of the hard models were adopted as about 1.1~1.5 times those for the soft models in lamellar β grains. On the basis of the size-dependent relationship of CRSS [13,17], CRSS values of the **a_1_** system, **a_2_** system, and **a_3_** system on the basal and prismatic planes were adopted to be the ratio of 1: 1.33: 1.41 based on the size of secondary α grains of the TC11 alloy. The values of the remaining slip parameters were finally determined by comparing the simulation results with the experimental curves on the basis of a calibration process adopted by Hasija et al. [11].

## 4. Results and Discussions

**Figure 6:**
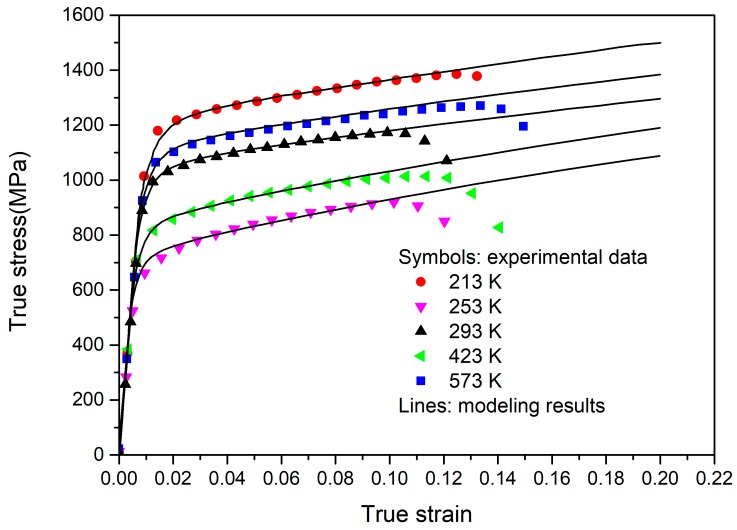
The compared results of numerical modelling with experiments.

**Figure 7:**
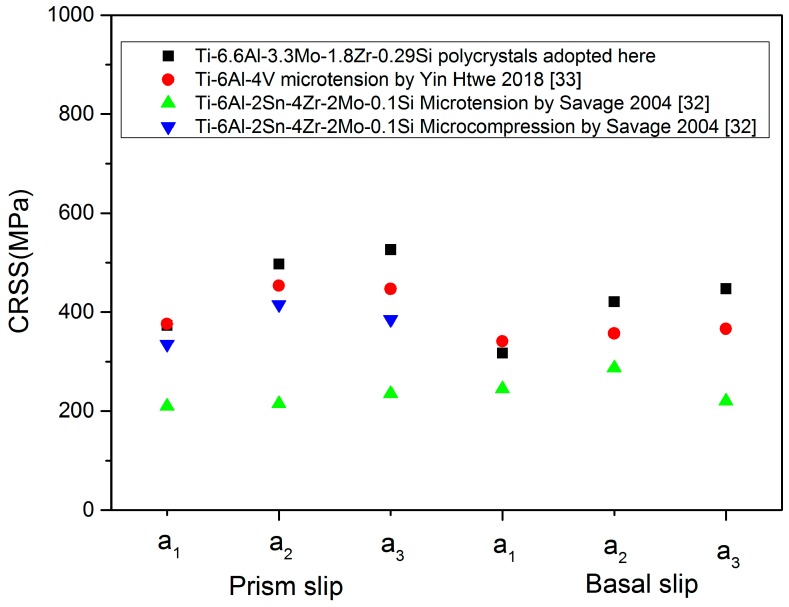
Summaries of the CRSS values for the TC11 alloy with those experimentally obtained values for a single α + β colony at room temperature [32,33].

The compared results of numerical modelling with the experiments are depicted in the temperature range from 213 to 573 K. As evidenced in Figure 6, the numerical results matched well with the experiments for all the temperatures. Such coincidence indicates that the current CPFE model has the capability to describe the tension behavior of the duplex TC11 alloy, including the linear-elasticity, nonlinear work-hardening at the initial yielding stage, and approximately linear hardening behavior at the large strain region. Nevertheless, some errors occurred at the instability stage. The main reason for this is that the damage failure was not considered in the current model. In the globular α grains, the values of CRSS at 293 K were 329 and 392 MPa for the <a>-slip systems on the prismatic and basal plane, respectively, and 874 MPa for the <c + a>-slip system on the pyramidal plane. Such CRSS values of the slip system were comparable to the values used in the polycrystalline modelling of a similar alloy [11,12,13,14]. Among the lamellar α grains, the determined CRSS values for the basal slip at room temperature were 317, 421, and 447 MPa and those for the prismatic slip were 373, 497, and 526 MPa. Figure 7 depicts the comparison of the values of CRSS for the TC11 alloy with those experimentally obtained values for a single α + β colony [32,33]. It was found that the anisotropic behavior in the α + β colony was well captured in the polycrystalline model of the duplex TC11 alloy, while the CRSS values adopted here were slightly higher than the experimentally obtained values for a single α + β colony. As illustrated by Jones [35] and Willians et al. [22], the CRSS values were sensitive to the stress-state and should increase with an increase in Al concentration of the titanium alloys [22]. In addition, the CRSS values were also dependent on grain size and the thickness of the lamellar grains [13,15]. Therefore, the higher values obtained here are suggested to be related to the more complex constraints among the neighboring grains, higher Al content, and the smaller size of the grains in the TC11 alloy. Besides the experiments, the temperature-dependent crystal plasticity model provided an alternative method to better understand the microstructure–property relationship. To further enhance the validity of the model, the deformation mechanism of the α/β grains with various microstructural morphologies should be accurately revealed in the future.

## 5. Conclusions

The flow behavior of a Ti-6.6Al-3.3Mo-1.8Zr-0.29Si alloy in a temperature range from 213 K to 573 K were studied by temperature-dependent crystal plasticity modelling. The experimental results indicate that the initial yield stress decreased as temperature increased and work-hardening was observed as temperature insensitive. The modified crystal plasticity formulas employed here considered the temperature effect on the values of initial critical shear stress. Good coincidence was obtained between the modelling and experimental results. The determined values of CRSS for the slip systems were comparable to the published data, indicating that the polycrystalline model has the capability to describe the observed temperature-dependent tensile properties within the investigated range of temperatures. The temperature-dependent crystal plasticity model will provide an alternative method to better understand the microstructure–property relationship in the future.

## Figures and Tables

**Table 1:** The parameters adopted in the model.

Constitutive Equations	Model Parameters			
**Elasticity**				
$T=C:E_{e}$ $C(T)=C_{\mathrm{RT}}\frac{\mu (T)}{\mu_{\mathrm{RT}}}$ μ(T)=49.02−5.821/[exp(181/T)−1]	*μ*_RT_ = 42 GPa; C_11_ = 162 MPa; C_33_ = 180 MPa; C_12_ = C_21_ = 92 MPa; C_13_ = C_31_ = C_23_ = C_32_ = 69 MPa; C_44_ = C_55_ = 46 MPa; C_66_ = (C_11_ − C_12_)/2 =35 MPa; Other = 0 [15]			
**Slip**				
${\dot{\gamma }}^{\left(i\right)}=\overset{}{{\dot{\gamma }}_{0}^{\left(i\right)}}{\left(\left|\frac{\tau^{\left(i\right)}}{\tau_{c}{}^{\left(i\right)}}\right|\right)}^{\frac{1}{m}}\mathrm{sgn}(\tau^{\left(i\right)})$	${\dot{\gamma }}_{0}^{\left(i\right)}=0.001\;s^{-1}$; m = 0.02 [11,18]			
${{\dot{\tau }}_{c}}^{\left(i\right)}=h_{0}{}^{\left(i\right)}\sec h^{2}\left|\frac{h_{0}{}^{\left(i\right)}\gamma }{\tau_{s}{}^{\left(i\right)}-\tau_{0}{}^{\left(i\right)}}\right|(\sum_{j}\left[q_{1}+(1-q_{1}\right)\delta_{ij}]{\dot{\gamma }}^{\left(j\right)}$ $\left|\tau_{0}{}^{\left(i\right)}(T)\right|=s_{a}^{\left(i\right)}+\overset{\wedge }{\tau^{\left(i\right)}}{\left[1-{\left(\chi \frac{k_{B}T}{\Delta G_{0}{}^{\left(i\right)}}\right)}^{1/q}\right]}^{1/p}$	q_1_ = 1, p = 0.5 and q = 2 for α grains; q_1_ = 1, p = 0.5 and q = 1.25 for β grains [8]			
	Globular α phase			
	Parameters	Basal<a>	Prism<a>	Pyr<c + a>
	$s_{a}^{\left(i\right)}\left(\mathrm{MPa}\right)$	246	230	610
	$\overset{\wedge }{\tau^{\left(i\right)}}\left(\mathrm{MPa}\right)$	788	765	1500
	$\Delta G_{0}{}^{\left(i\right)}\left(\mathrm{eV}\right)$	1.56	1.43	2.1
	$h_{0}{}^{\left(i\right)}\left(\mathrm{MPa}\right)$	200	160	440
	$\tau_{s}{}^{\left(i\right)}\left(\mathrm{MPa}\right)$	660	780	1600
	χ	20.1	23.1	28
	Lamellar α phase (Basal)			
	Parameters	a_1_	a_2_	a_3_
	$s_{a}^{\left(i\right)}\left(\mathrm{MPa}\right)$	195	259	297
	$\overset{\wedge }{\tau^{\left(i\right)}}\left(\mathrm{MPa}\right)$	650	862	988
	$\Delta G_{0}{}^{\left(i\right)}\left(\mathrm{eV}\right)$	1.43	1.43	1.43
	$h_{0}{}^{\left(i\right)}\left(\mathrm{MPa}\right)$	140	187	218
	${\tau_{s}}^{\left(i\right)}\left(\mathrm{MPa}\right)$	560	740	855
	χ	23.1	23.1	23.1
	Lamellar α phase (Prism)			
	Parameters	a_1_	a_2_	a_3_
	$s_{a}^{\left(i\right)}\left(\mathrm{MPa}\right)$	209	277	294
	$\overset{\wedge }{\tau^{\left(i\right)}}\left(\mathrm{MPa}\right)$	670	889	943
	$\Delta {G_{0}}^{\left(i\right)}\left(\mathrm{eV}\right)$	1.56	1.56	1.56
	$h_{0}{}^{\left(i\right)}\left(\mathrm{MPa}\right)$	160	216	234
	${\tau_{s}}^{\left(i\right)}\left(\mathrm{MPa}\right)$	660	883	890
	χ	20.1	20.1	20.1
	Lamellar β phase ({110}<111>)			
	Parameters	Soft	Hard	
	$s_{a}^{\left(i\right)}\left(\mathrm{MPa}\right)$	239	358	
	$\overset{\wedge }{\tau^{\left(i\right)}}\left(\mathrm{MPa}\right)$	778	1167	
	$\Delta G_{0}{}^{\left(i\right)}\left(\mathrm{eV}\right)$	1.34	1.34	
	${h_{0}}^{\left(i\right)}\left(\mathrm{MPa}\right)$	165	250	
	${\tau_{s}}^{\left(i\right)}\left(\mathrm{MPa}\right)$	660	990	
	χ	24.1	24.1	
